# Accuracy of ct evaluation for cervical spine clearance in the ground level fall population – a retrospective cohort study

**DOI:** 10.1186/s12873-022-00657-x

**Published:** 2022-06-11

**Authors:** John Culhane, Alan Parr, Philippe Mercier

**Affiliations:** 1 Saint Louis University School of Medicine, Saint Louis, MO USA; 2 Departments of Trauma and Neurosurgery, Saint Louis University, Saint Louis, MO USA; 3 Department of Surgery, Saint Louis University, 1008 Spring Ave, Saint Louis, MO 63110 USA; 4 Department of Neurosurgery, Saint Louis University, 1008 Spring Ave, Saint Louis, MO 63110 USA

**Keywords:** Ground Level Fall, Cervical Spine, Clearance, Elderly, Trauma

## Abstract

**Background:**

Clinically occult cervical spine (CS) injuries are well described in blunt trauma, however delay in identifying these injuries and clearing the CS can result in morbidity. Our study examines the ground level fall (GLF) population to analyze whether computed tomography (CT) alone can rule out unstable injury in this group with lower force mechanism.

**Methods:**

This is a single center, retrospective cohort study. All GLF patients in the institutional trauma registry between 6/1/2012 through 12/31/2019 were included. These comprise all trauma patients evaluated in the emergency department with Injury Severity Score (ISS) > 0, including both activations and consults with both clinical and radiological spine evaluation. Patients who could not be cleared by National Emergency X-ray Utilization Study (NEXUS) criteria underwent CT. Patients with CT or clinical suspicion of cord or ligamentous injury underwent MRI. CT occult injuries were identified by MRI and clinical exam, with MRI identifying all unstable injuries.

**Results:**

Sixty-nine (2.0%) of patients had CS injury without acute CT abnormality. Of these, 11 (0.3%) required surgery and were considered unstable. All patients who required surgery had a neurologic deficit. Negative predictive value (NPV) of CT for unstable CS injury was 99.7%. The combination of acute CT findings and neurologic deficit ruled out unstable CS injury with 100% NPV.

**Conclusion:**

In the GLF population, CT alone rules out unstable CS injury with high, but not perfect NPV. The combination of absence of acute CT findings and acute neurologic deficits rules out unstable CS injury with 100% NPV.

**Supplementary information:**

The online version contains supplementary material available at 10.1186/s12873-022-00657-x.

## Background

Cervical spine (CS) injuries are common in blunt trauma. A recent review showed an incidence of 3.7% among all trauma patients. Those without accurate clinical exam had an even higher rate of 7.7% [1]. Cervical spine injuries are not always clinically apparent initially, but failure to recognize them may have devastating consequences. A combination of history, clinical exam, and usually imaging is required to identify unstable injuries that require treatment [2].

Ground level falls (GLF) are a common mechanism of trauma. They are generally lower force than mechanisms such as motor vehicle collisions (MVC), but they can still result in serious injury. They often affect the elderly who are more fragile and thus especially vulnerable to musculoskeletal injury [3]. Chronic pain, degenerative disease and mental status changes can make clinical clearance of elderly GLF patients challenging.

When the CS cannot be cleared by clinical criteria, the consensus next step is to obtain a CT scan of the CS [4]. There is controversy regarding what to do if the CT shows no acute injury, but the patient has persistent cervicalgia, or cannot cooperate with a clinical exam due to obtundation or intubation. Recommendations include clearing the CS based on the CT, maintaining the cervical collar (C-collar) and some degree of activity restriction until later re-examination, or obtaining a Magnetic Resonance Image (MRI).

During the time prior to CS clearance, often while waiting for an MRI, the patient continues to wear a collar and may be restricted to bedrest. Collars and immobility may have adverse effects on trauma patients, especially the elderly [5]. The purpose of our study is to determine whether the GLF population is safe to clear based on CT alone, thus avoiding the additional step of an MRI. The questions we seek to answer are: given the low force nature of GLF, are ligamentous disruptions without CT detectable bone injury common enough to warrant investigation? In the elderly CS, would fragile bones break first under a stress great enough to rupture ligaments, rendering CT more sensitive? Alternatively, does weakened connective tissue in the elderly make this population more vulnerable to unstable occult as well as clinically overt CS injury? To help answer these questions, we present a series of ground level fall patients to assess the incidence of CT negative CS injuries in this special population.

## Methods

This is a single institution, retrospective cohort study from a level I trauma center with a prospectively maintained trauma database. Every effort was made to follow best practices for chart review as described by Kaji [6]. Chart abstraction was performed by 6 full-time registrars monitored by a senior clinical data abstractor. The senior abstractor ensures inter-rater reliability and compliance with specific protocols to meet National Trauma Data Bank and Trauma Quality Improvement Program standards. The abstractors maintain the registry prospectively for all trauma patients; thus blinding was not required because abstraction of data took place prior to the inception of this study. The registrars examined all encounters present in the chart at the time of abstraction for evidence of delayed presentation of injury. This includes radiologic reports and clinical interactions both for the index admission and subsequent follow-up visits.

The only missing data were complete details for some of the outside CT scans, which only affected the evaluation of chronic degenerative changes. The presence or exclusion of acute injury was available for all scans. Any data unavailable from the registry was retrieved by direct chart review by one of the investigators.

All GLF patients in the institutional trauma registry between 6/1/2012 through 12/31/2019 were included. These comprise all trauma patients evaluated in the emergency department with Injury Severity Score (ISS) > 0, including both activations and consults. Our trauma center uses three activation levels: I, II and III, in descending order of severity. All are based on standardized criteria, but there is greater subjectivity involved in assigning a trauma to Class III (consult), which relies more on the discretion of the emergency department physician. Typically, the trauma service is consulted for any trauma patient with injuries serious enough to require admission. Trauma activation criteria are included in supplemental file number 1.

**Fig. 1:**
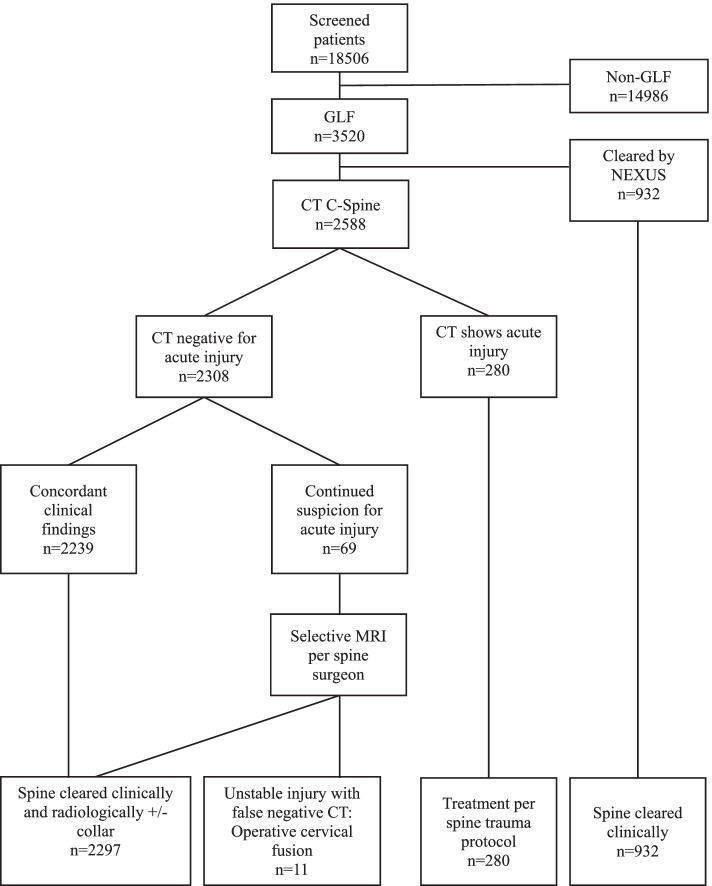
Selection and evaluation of GLF cohort

This cohort includes all GLF patients with at least one injury captured by the ISS categorization. It does not include all patients who were evaluated because those without injury are not entered into the registry. Per our standard practice, patients were evaluated for initial clinical C-spine clearance by NEXUS criteria. Patients who did not meet the NEXUS criteria underwent CT. Patients with CT or clinical suspicion of cord or ligamentous injury underwent MRI. CT occult injuries were identified by MRI and clinical exam. Figure 1 illustrates the selection and evaluation of the GLF cohort.

Further patient characteristics and management outcomes were chart reviewed. For the GLF mechanism we excluded contact sports, Hoyer lift accidents, impact from falling objects, jumping over fences, falls down stairs and slopes, and falls from horses, inversion tables, and moving vehicles. We included falls from standing, including those due to seizures, falls out of bed other than the top bunk, falls from wheelchairs, other chairs, and scooters, falls getting into or out of bathtubs and stationary vehicles, and falls in which the patient struck an object such as furniture. CS injury was defined as any injury to the CS identified by International Classification of Diseases 9 (ICD9) and ICD10 diagnosis codes. Baseline characteristics were compared for GLF patients versus all other mechanisms. A subset of patients was identified who had a final diagnosis of cervical spine injury but no acute findings on CT scan of the CS. We labelled these CT negative injuries (CTNI). These represent false negative CT evaluation. MRI findings and treatment were reported for these patients.

Significance for categorical data was calculated with Chi Square. Student’s t-test was used for continuous data and Mann–Whitney U test was used for ordinal data. Binary classification tests (sensitivity etc.) with 95% confidence intervals were calculated using a generalized linear regression model with binomial distribution and identity link. All statistical analyses were conducted on IBM SPSS Statistics for Windows, version 26.0 (IBM Corp., Armonk, N.Y., USA).

## Results

There were 18,506 trauma patients treated at Saint Louis University (SLU) between the dates 6/1/2012 through 12/31/2019. Three thousand five hundred twenty patients had a GLF. Of these, 104 (3.5%) were activation level I; 769 (21.8%) were level II; and 2647 (75.2%) were level III. Two thousand five hundred eighty-eight patients underwent CT of the CS, with the rest cleared by NEXUS clinical criteria. A comparison of baseline characteristics between the population with GLF versus other mechanisms shows that the GLF population is much older with a greater proportion of female patients. Other demographic and injury characteristics were roughly similar. (Table 1).

**Table 1:** Baseline characteristics

**Characteristics**	**Non-GLF**		**GLF**		
	***n***	**%**	***n***	**%**	***p***
**All**	14,986	80.9	3520	19.1	
**Sex—Female**	3712	24.8	1874	53.2	< 0.001
**Patients with C-spine Injury**	1713	11.4	349	9.9	0.029
**Total Injuries**	2483		543		
**Types of C-spine Injury**					
** Fracture**	1610	64.8	361	66.5	< 0.001
** Dislocation**	84	3.4	16	2.9	0.69
** Cord Injury**	144	5.8	48	8.8	< 0.001
** Central Cord Syndrome**	40	1.6	21	3.7	< 0.001
** Sprain**	645	26.0	97	17.9	0.24
** Multiple Injuries**	478	19.3	114	21.0	0.9
	**Non-GLF**			**GLF**	
	**mean**			**mean**	***p***
** Injury Severity Score**	12.19			9.93	< 0.001
** Total GCS**	13.7			14.22	0.077
** Age**	41.34			68.3	< 0.001
** BMI**	28.85			28.1	0.047

In the GLF group, 349 (9.9%) patients with a mean age of 72.2 had a total of 543 C-spine injuries. Of these, 272 (7.7%) patients had a total of 361 C-spine fractures, all identified on CT. This leaves 77 (2.2%) patients with a C-spine injury without a fracture. Eight of these patients had a total of 10 other acute findings on CT. (Table 2), for a total of 280 patients with CT positive C-spine injuries.

**Table 2:** Acute cervical spine ct findings other than fractures

CT Finding	*n*
Soft tissue edema	3
Disk space widening	2
Misalignment of vertebrae, (anterolisthesis or subluxation)	3
Widened atlantodentate interval	1
Widened predentate interval	1

Of the 8 patients with another acute finding on CT, 6 underwent MRI. All 6 were found to have ligamentous injury. Sixty-nine (2.0%) patients had a final diagnosis of C-spine injury with no acute abnormality on C-spine CT. Eighteen of these patients underwent MRI. Four showed no signs of acute trauma. The other 14 patients had the following 27 findings, with some patients showing multiple findings. (Table 3).

**Table 3:** C-spine MRI findings with CT scan negative for acute injury

Acute MRI Finding	*n*
Cord Contusion/Edema	6
Cord Compression	3
Ligamentous injury	3
Disc herniation	1
**Chronic MRI Finding**	***n***
Stenosis	12
Congenital short pedicles	1
Post-operative changes	1

Fifty-eight patients showed chronic changes on CT. Four showed no chronic changes. Chronic changes for 7 were unknown because complete results of scans from an outside hospital were not available, only a reference to the acute abnormality in progress notes. All 9 CTNI patients with available complete CT results who required surgery had chronic degenerative changes on CT.

The 69 patients with C-spine injuries with CT scan negative for acute injury were ultimately found to have the following 71 diagnoses based on MRI and clinical assessment. Two patients had more than one diagnosis: (Table 4).

**Table 4:** C-spine injuries with CT scan negative for acute injury

C-spine Injury	*n*
Cervical dislocation:	3
Central Cord Injury:	10
Other Cervical Cord Injury:	2
Neck Sprain:	56

Initial Glasgow Coma Scale (GCS) for the GLF group was 15 for 2709 (76.9%), 14 for 811 (23.0%), and 13 or less for 342 (9.7%). GCS for the CTNI group was: 15 for 59 (85.5%), and 14 or less for 10 (14.5%). Among patients with GCS of 13 or less, 3 (4.3%) had a CTNI (false negative CT) for stable injury and zero had a CTNI for unstable injury. Thirteen (18.8%) of the CTNI patients had a neurologic deficit. Of the 3 that had ligamentous injury on MRI with no acute abnormality on CT, all were paralyzed on presentation.

Treatment results: 36 (52.2% of CTNI) patients were cleared with no treatment. Twenty-two (31.9% of CTNI) were cleared with a cervical collar. Eleven (15.9% of CTNI) patients with a mean age of 64.0 underwent spinal fusion. These 11 (0.31% of the total GLF population) patients represent the group with false negative CT results for unstable injury. Six of these 11 patients were under the age of 65.

Of those who underwent surgery, all except one had a neurologic deficit. The patient who did not have a neurologic deficit had an MRI showing stenosis with possible cord contusion, but no disc or ligamentous injury. He was initially sent home in a collar. He underwent surgery 5 weeks later due to intractable pain. Thus, all patients who required urgent surgery had a neurologic deficit. For the population of GLF patients, CT correctly identified C-spine injuries requiring treatment with the following binary classification characteristics. (Table 5).

**Table 5:** Accuracy of CT scan prediction of C-spine injuries requiring treatment

	**All Patients**			
	**Surgery**	**95% CI**	**Surgery or Collar**	**95% CI**
**Sensitivity**	0.804	(.682, .897)	0.748	(.671, .816)
**Specificity**	0.936	(.927, .943)	0.952	(.944, .959)
**PPV**	0.155	(.115, .202)	0.383	(.325, .442)
**NPV**	0.997	(.995, .998)	0.99	(.986, .993)
	**Patients with GCS > 13**			
	**Surgery**	**95% CI**	**Surgery or Collar**	**95% CI**
**Sensitivity**	0.796	(.677, .889)	0.75	(.672, .819)
**Specificity**	0.936	(.927, .944)	0.953	(.945, .960)
**PPV**	0.178	(.133, .229)	0.409	(.348, .472)
**NPV**	0.996	(.994, .998)	0.989	(.984, .992)

The combination of acute CT changes and neurologic abnormality had a sensitivity and negative predictive value of 100% for injury requiring urgent surgery.

## Discussion

Unrecognized or undertreated CS injury can lead to devastating consequences including quadriplegia, yet despite extensive study, there is still no consensus on the exact method of CS clearance after trauma [2]. CT scans have become the standard of care in evaluating the CS in those for whom imaging is required [7]. Providers face a dilemma when the CT scan shows no acute injury, but the clinical exam is unreliable, or the patient has persistent cervicalgia. There is considerable debate regarding the need for MRI to clear the CS in this situation. If we could identify a subset of trauma patients at lower risk for occult unstable CS injuries that clinicians would be more comfortable clearing both clinically and based on CT, we could potentially conserve resources and spare these patients the morbidity of prolonged immobilization.

GLF is a mechanism that is both very common and low force. GLF resulted in 2.1 million ED visits in the US in patients older than 65, a Fig. 10 times greater than that of motor vehicle crashes. One in 3 geriatric patients suffers a GLF each year [8]. GLF are most common among the elderly. Patients in a recent study of spinal fractures due to falls from standing had an average age of 76.6 [9]. In a study of GLF, one must consider not only the mechanism, but also the special characteristics of the typical elderly trauma patient when assessing a protocol for CS clearance.

While the forces involved in GLF are lower than those of MVC, the mechanism is not innocuous. Our data show that the Injury Severity Score (ISS) is significantly lower for GLF versus other types of trauma at 9.93 versus 12.19, but an ISS of nearly 10 still represents clinically important injury. The percent of patients with a CS injury was only slightly lower, with an absolute difference of just 1.2%. The incidence of fracture and cord injury were higher for GLF, and the incidence of central cord syndrome was over twice as high. This is consistent with other reports. In a study of ground level falls recorded in the National Trauma Data Bank, Spaniolas et al. found a median ISS of 8 and a mortality of 3.2%. The mortality among those over 70 was 4.4% versus 1.6% for younger patients [3]. Hall et al. found an incidence of 229/1408 spine fractures due to falls from standing height. One hundred forty of these were cervical. Forty three of 229 (18.7%) spine fracture patients died [9]. This shows that GLF is not a benign mechanism. The force of a ground level fall is sufficient to inflict serious injury, especially for older patients.

GLF patients may not always have a reliable clinical exam. Our group included 811 patients (23.0%) with GCS < 15. In standard clinical practice, CS clearance would be delayed for most if not all of them. For the overall trauma population, there is disagreement regarding whether a high-quality CT scan is sufficient to rule out unstable CS injury in the absence of a reliable clinical exam, or in a patient with cervicalgia. Some studies show that a negative CT is sufficient. Inaba et al. performed a multi-institutional prospective trial of 10,276 patients who could not be cleared clinically due to unreliable exam, CS tenderness, or neurologic symptoms. There were 3 false negative CT scans, which yielded an NPV of 99.97% for clinically significant injuries. These 3 patients had acute neurologic deficits, thus the combination of normal neurologic exam and negative CT resulted in an NPV of 100% [10].

In a systematic review forming the basis of the 2015 Eastern Trauma Association guidelines for the clearance of the CS in obtunded patients, 1017 obtunded patients in 5 studies showed no neurologic change after collar removal based on negative CT. One thousand seven hundred eighteen patients in 11 studies showed 161 (9%) stable injuries with no unstable CS injury. NPV was 91% for stable, and 100% for unstable injuries found on subsequent MRI and clinical follow up. The authors considered the risk of false positive MRI results leading to unnecessary treatment, risk of transport and time outside ICU, and the cost of MRI in formulating their conditional recommendation to remove the C-collar after a negative high-quality CT. The authors acknowledge that this policy may lead to a “non-zero rate of neurologic deterioration” [4].

Other studies show a higher false negative rate for CT CS evaluation in trauma. A literature review by Malhotra in 2017 showed 4/3370 obtunded patients with unstable CS injury after negative CT versus 10/1387 alert, awake patients [11]. A subsequent study by the same author analyzed 1080 trauma patients who received a CT and then a follow up MRI within 48 h. Of 712 patients with a negative CT, 149 had positive findings on MRI, 97 of which were ligamentous and cervical fascial injuries. Sixty-five of the 149 had experienced a fall from standing. One out of 65 injuries was unstable. CT had a 98.5% NPV, and 0.42% of patients had a change in management due to MRI findings [12].

False negative evaluation can be reduced by more testing, but the increased sensitivity comes with a cost. More evidence has accumulated documenting the harm of extended CS work-up and precautions. Peck showed that C-collars in the elderly are associated with pressure sores, elevated intracranial pressure, respiratory compromise, swallowing difficulty, delirium, and difficulty with mobility [5]. Dunham et al. conducted a literature review of comatose blunt trauma patients with negative CS CT scans. They found a risk of 2.5% of CS instability. However, they also found risks of 26.2% of ICU complications of prolonged C-collar use, 9.3% to 14.6% of secondary brain injury during MRI transport and 20.6% risk of aspiration during MRI scanning [13]. Bedrest in trauma patients has been associated with complications of immobility such as muscle weakness, pressure ulcers and deep vein thrombosis [14]. There are financial costs as well. A 2021 study showed that the average cost of negative MRI plus waiting time following negative CT was 4628 dollars [15]. The risk of missing an unstable injury must be balanced against the cost and risk of immobilization and further workup.

Despite an increasing number of studies demonstrating safety, there is still widespread resistance to clearing the CS based on CT. These results may help explain why. Although neurologic deficit identified all patients needing urgent surgery in this study, patients do not always have an accurate neurologic exam. For instance, in our cohort, 9.7% of the patients had a GCS of 13 or less. If one of these patients had been obtunded enough to mask the neurologic deficit, clearing the CS based on CT without obtaining an MRI would have missed the injury.

Limitations: Our registry includes only patients with ISS > 0, rather than all patients with a GLF. C-spine injuries may be over-represented in our cohort versus one that includes GLF patients with a completely negative work-up.

## Conclusions

The risk of a CS injury following a ground level fall is similar to that of trauma from higher force mechanisms. It is possible to have an isolated CS ligamentous injury from a ground level fall with a false negative CT. We cannot say that the bone is weaker than the ligament and will always break first, thus the absence of fracture does not rule out unstable CS injury. The combination of acute CT findings and neurologic deficits did detect all patients who needed urgent surgery, but we do not always have an accurate neurologic exam. The GLF mechanism is not low enough risk to exclude clinically significant CS injuries with CT alone. This does not mean that more liberal use of MRI is necessarily beneficial, but rather that the risks of an MRI based clearance protocol must be balanced against the non-zero rate of false negative CT. Only a large, randomized trial of a clearance protocol based on CT alone versus CT followed by MRI with the endpoint of neurologic deterioration could answer this question definitively. In the meantime, we can reduce diagnostic uncertainty by developing a more rigorous reference standard for spinal instability and standardization of indications for surgery. Clinicians can accept a consensus recommendation of a highly sensitive protocol as standard of care recognizing that no protocol will ever be perfect.

## Supplementary information

**Additional file 1.**

## Data Availability

The datasets analyzed during the current study are not publicly available because the data are part of the patients’ medical record and are treated as confidential. A completely de-identified version of the data is available from the corresponding author on reasonable request, following approval of the institutional review board.

## Abbreviations

CS: Cervical Spine
CT: Computed Tomography
CTNI: CT Negative Injuries
GCS: Glasgow Coma Scale
GLF: Ground Level Fall
ICD9-10: International Classification of Diseases-9,10
ISS: Injury Severity Score
MRI: Magnetic Resonance Imaging
MVC: Motor Vehicle Collisions
NEXUS: National Emergency X-ray Utilization Study
NPV: Negative Predictive Value
SLU: Saint Louis University
