# Supplementary material for: Accuracy of ct evaluation for cervical spine clearance in the ground level fall population – a retrospective cohort study
Source: BMC Emerg Med. 2022 Jun 11;22:106. doi: 10.1186/s12873-022-00657-x (PMC9188238; doi:10.1186/s12873-022-00657-x)
Supplement: Supplementary file 1 — Additional file 1. [file 12873_2022_657_MOESM1_ESM.pdf]

Attachment B

SLU Hospital TRAUMA TEAM ACTIVATION CRITERIA

Trauma Activation Page: Please include Class (1 or 2) trauma, age, gender, MOI, VS, ETA

CLASS 1 TRAUMA<br>(Full Trauma Team)	CLASS II TRAUMA<br>(Partial Trauma Team)	CLASS III TRAUMA<br>(Evaluation)
**AIRWAY/BREATHING ☐**<br>Airway Compromise<br>☐ Intubated patients from scene<br>☐ Respiratory compromise (including patients transferred from OSH with ongoing compromise)<br>☐ Need of emergent airway<br>☐ Respiratory rate <10 or >29/minute	**AIRWAY/BREATHING**<br>☐ Decreased breath sounds (suspect hemo/pneumothorax)<br>☐ Known Pneumothorax/Hemothorax	**AIRWAY/BREATHING**<br>☐ Normal<br>☐ No abnormalities
**CIRCULATION**<br>☐ Confirmed SBP ≤90 at any time<br>☐ Confirmed SBP ≤ 100 if ≥ 65 years old<br>☐ Active or uncontrolled hemorrhage<br>☐ Receiving blood to maintain VS	**CIRCULATION**<br>☐ Normal Vital Signs<br>☐ Controlled bleeding without tourniquet	**CIRCULATION**<br>☐ Normal with stable vital signs<br>☐ No hemodynamic issues
**DISABILITY**<br>☐ GCS ≤ 13 attributed to trauma<br>☐ Acute onset motor/sensory loss due to injury (suspected SCI).	**DISABILITY ☐**<br>GCS 13-14 attributed to trauma<br>☐ Open or depressed skull fracture<br>☐ LOC > 1 minute	**DISABILITY**<br>☐ GCS 15<br>☐ No or brief LOC (< 1 minutes)
**CHEST/ABDOMEN/PELVIS**<br>☐ ED physician's discretion	**CHEST/ABDOMEN/PELVIS**<br>☐ Sub-Q emphysema/crepitus without airway compromise<br>☐ Chest wall instability/deformity or crepitus without airway/respiratory compromise<br>☐ Abdomen firm, tender and/or distended<br>☐ Seatbelt<br>☐ Known/suspected solid organ injury<br>☐ Known/suspected Pelvic fracture<br>☐ Pregnancy with abdominal pain after traumatic event	**CHEST/ABDOMEN/PELVIS**<br>☐ Superficial abrasions and contusions<br>☐ No abnormalities
**EXTREMITIES**<br>☐ Extremity trauma with loss of distal pulse<br>☐ Tourniquet in place for uncontrolled bleeding<br>☐ Amputation proximal to wrist<br>☐ Amputation proximal to ankle	**EXTREMITIES**<br>☐ Neurovascular compromise to extremity<br>☐ Two or more long bone fractures (humerus, femur, tibia)<br>☐ Open fractures of long bone(s)<br>☐ Penetrating injury to extremity distal to pelvis or axilla<br>☐ Crushed, degloved, or mangled extremity	**EXTREMITIES**<br>☐ Isolated fracture of extremity without neurovascular compromise<br>☐ Crush injuries or amputation to digits
**MOI**<br>☐ Penetrating injuries to the head, neck, torso, abdomen, or pelvis/groin<br>☐ Pelvic fractures/unstable pelvis with hemodynamic compromise<br><br>**Other ☐**<br>EM Physician discretion	**MOI**<br>☐ Falls greater than >12 feet/steps<br>☐ Penetrating to head, face, torso or extremities with no hemodynamic, neurological, or respiratory compromise<br>☐ High-risk MVC with:<br>Intrusion of vehicle >12" in occupant compartment<br>○ Ejection (partial or complete) from vehicle ○ Rollover<br>○ Death in same passenger compartment ○ > 40 mph<br>○ Extrication time > 20 minutes<br>☐ MCC or ATV crash >20 mph<br>☐ MCC or ATV crash at any speed for patients ≥ 65 years old<br>☐ High-energy dissipation or rapid decelerating incidents including:<br>○ Ejection from motorcycle, ATV, animal, etc.<br>○ Striking fixed objects with momentum<br>○ Blast or explosion<br>☐ Auto-pedestrian/auto-bicycle with (> 5 mph) impact<br>☐ Burns >10% TBSA (2nd or 3rd degree burns) with associated injuries<br>☐ High voltage electrical injury with stable VS<br>☐ Emergency Medicine Physician discretion	**MOI**<br>☐ Injured patients that do not meet either Class I or Class II activation criteria<br>☐ Single-system injuries with low concerns for occult or multisystem trauma.<br>☐ Falls ≤ 12 feet/steps<br>☐ Emergency Medicine physician may consult the Trauma Surgeon/sub-specialty service as needed.

- UPGRADING:
  - May occur at the discretion of the ED or Trauma Surgeon based upon patient's physiologic condition upon presentation ○ Upgrading must be activated in the paging/notification system.
  - Time and level of upgrade should be documented in the trauma narrator.
- There should be NO DOWNGRADING. Once the team has been activated, team members may be dismissed at the discretion of the ED or Trauma attending when upon initial evaluation the patient failed to meet activation criteria.
- Activation time and arrival time for trauma team members should be documented in the trauma narrator.
- Arrival time for specialty service consults should be documented in the trauma narrator.
